# Evaluating Fertilizer Use Efficiency and Spatial Correlation of Its Determinants in China: A Geographically Weighted Regression Approach

**DOI:** 10.3390/ijerph17238830

**Published:** 2020-11-27

**Authors:** Xiuguang Bai, Tianwen Zhang, Shujuan Tian

**Affiliations:** 1 College of Economics and Management, Northwest A&F University, Yangling 712100, China; Morgen19ztw@outlook.com; 2 College of Horticulture, Northwest A&F University, Yangling 712100, China; tianshujuan@nwafu.edu.cn

**Keywords:** fertilizer use efficiency, spatial effect, geographical weighted regression, impact factors, stochastic frontier production function

## Abstract

Improving fertilizer use efficiency (FUE) is an effective means to reduce fertilizer use and environmental contamination. Few studies have considered the spatial effects of FUE and its determinants. This paper calculated the FUE of agricultural production by adopting panel data on 31 provinces in China from 2007 to 2017 using a stochastic frontier method with a heteroscedastic inefficiency term, and discussed the spatial characteristics. Further, the geographical weighted regression model (GWR) was employed to examine the spatial impact of factors on FUE and revealed the spatial dispersion and agglomeration effect. The results show that averaged FUE in China was 0.722, and had a significantly decreasing trend with a significant regional difference and spatial positive correlation in different provinces. The non-agricultural employment ratio was the leading factor for increasing FUE, and its degree of influence showed a decreasing trend from eastern to western China. The different agricultural industry development modes, crop planting patterns adjustment, labor transfer, and policy incentive systems for increasing the non-agricultural employment ratio should be developed for different regions. Farmers’ income had a negative impact on FUE, but the influence degree decreased annually. Education level had a negative impact on FUE and was relatively weak, but the influence degree was increasing. This should strengthen the exploration of a scientific and practical technical training system for farmers on fertilizer use while improving educational levels in different regions on the basis of local characteristics. The impact of disasters on FUE depended on their severity, and a combined weather and disaster forecasting mechanism should be developed.

## 1. Introduction

Chemical fertilizer is a crucial input for crop production, and has made a substantial contribution to grain yields, especially in China, where chemical fertilizer has contributed to 56.81% of the increase in grain yield [1,2]. The application of chemical fertilizer in China has increased from 8.84 million tons in 1978 to 56.53 million tons in 2018. However, increasing fertilizer use has weakened the marginal effect of the increasing yield [3], and overuse has resulted in low fertilizer use efficiency (FUE) [4,5] and various environmental problems, such as acid rain [6], soil pollution [7], biodiversity loss [8], and water eutrophication and contamination [9,10], which threaten the sustainable development of agriculture and even public health [11]. To put into practice the conviction that clear waters and green mountains are as valuable as mountains of gold and silver, and to achieve green agricultural production and sustainability, it is necessary to reduce the unreasonable use of chemical fertilizers.

Efficient management of agricultural chemical fertilizer by improving FUE or fertilizer productivity is a critical measure to reduce fertilizer use [5]. Especially in China, FUE is only approximately 30%, almost half that of FUE in developed countries [12]. Fertilizer use can be reduced by 50% if China were to increase the current FUE to the level of developed countries, without crop yield reduction [13,14], which shows great potential to reduce fertilizer use and eliminate the adverse environmental effect of chemical fertilizer. Therefore, it is worthwhile to examine the FUE of agriculture production in China and its influencing factors to improve FUE.

Considerable efforts have been made by the government for reducing fertilizer use and increasing FUE. The Chinese government proposed the Chemical Fertilizer Use Zero Increase by 2020 plan in 2015, and the Chemical Fertilizer Use Negative Increase plan was proposed in 2019, while the objective of implementing these policies is to increase FUE [5,12]. Around this goal, each province has formulated various policies and actively adjusted the agricultural industrial structure. Due to the different natural endowments and policies in each province, FUE is usually different in each province, while similar characteristics in adjacent regions and provinces may lead to spatial correlation and the agglomeration effect of FUE, so it is essential to analyze the spatial effects of FUE and its influencing factors.

Although current studies have already analyzed FUE and its determinants, few studies have considered the heteroscedasticity of error (the error usually assumed to have the same variance given any value of the independent variable; if the variance is different, the error is heteroscedastic) or have analyzed the spatial correlation and agglomeration effect of FUE, and the spatial effects of factors affecting FUE. This paper attempted to analyze the spatial effect of the factors influencing FUE at the provincial level using the geographical weighted regression (GWR) model on the basis of investigating the spatial correlation and agglomeration effect of FUE. This paper is innovative from various points of view. First, previous studies on FUE using the stochastic frontier analysis (SFA) model neglect the heteroscedasticity of error components, so the heteroscedasticity in both error terms and the stochastic and inefficiency error components are considered with the SFA model in this paper, which allows us to consider the impact of provincial characteristics on the efficiency, such as economic development level and average education degree. Second, although some studies have analyzed the spatial heterogeneity of FUE, few studies have considered the spatial correlation and agglomeration effect of different provinces. Because of the similarity of climate, resource endowment, economic development, and technology spillover in adjacent regions, neighboring provinces have obvious spatial correlation. Spatial correlation and agglomeration can significantly affect FUE in adjacent provinces. Third, studies have mainly used the Tobit model to measure the impacts of factors on FUE [5,15,16]. The spatial factor has not been considered, which can lead to estimation bias, and the corresponding results and recommendations may be misleading. Therefore, this study aimed to reveal the spatial distribution characteristics of FUE and analyze the spatial effects of factors affecting FUE. Using the spatial panel data from 2007 to 2017, this study calculated FUE and examined its spatial effect, then analyzed its determinants using the GWR model. Policy suggestions are put forward to provide a reference for improving FUE and reducing fertilizer use in agricultural production, which is crucial to the sustainable development of agriculture.

The rest of the paper is as follows. Section 2 presents the literature review of FUE and its influencing factors. The calculation method, model, and data sources are described in Section 3. Section 4 presents the empirical results of FUE and its influencing factors calculated by the GWR model. Section 5 discusses the findings, and Section 6 ends with conclusions and policy implications.

## 2. Literature Review

In this section, the conception and estimation method of FUE are first introduced, then the factors affecting FUE are discussed, followed by the methods and their advantages in estimating the impact of factors on FUE. It concludes with highlights on how this paper fills the gaps in the literature.

FUE is a main indicator for evaluating the effective use rate of fertilizer, and is usually used to address environmental pollution caused by fertilizer input [13]. Many studies have analyzed FUE from the perspective of natural and social sciences based on agronomy and economic theory. In natural sciences, the analysis of FUE mainly focuses on nitrogen use efficiency (NUE), which is defined as the ratio of nitrogen uptake to total nitrogen fertilizer input [13,17], and it is calculated commonly through field experiments. Duan et al. [6] investigated the effects of various fertilization experiments on NUE at four sites in China and found that manure treatment had the highest NUE for wheat and corn. Ladha and Chakraborty [17] indicated that the average world NUE is only 47%. Yang et al. [4] evaluated NUE based on a field experiment and showed that NUE decreases linearly with the increasing rates of nitrogen use in the Loess Plateau of China. Cao et al. [9] indicated that an active canopy sensor-based precision N management (CS-PNM) strategy increases NUE by 68–123% using a field experiment conducted in China. Wu et al. [12] calculated and compared the NUE of grain production in China and other countries in the world and showed that the NUE was approximately 30% in China, significantly lower than the world average of 50%, and that the NUE of different continents differs. Furthermore, Lassaletta et al. [13] showed that FUEs differ in different countries by using a 50-year data analysis, and compared to no straw treatment, the FUE increased by 10.57–48.77%, with a buried straw layer under lower nitrogen and irrigation levels [18]. Liang and Shi [19] showed that poly-Y-glutamic acid treatments significantly enhanced nitrogen and phosphorus uptake efficiency by 20.7–82.8% and 4.2–50.0% compared with no treatment, respectively.

Experimental methods have usually been used to calculate FUE in the natural sciences, and there may be rigorous experimental conditions that have neglected human adaptive activities; thus, it is not suitable for large-scale evaluation of FUE [20]. Therefore, many studies have evaluated FUE from the perspective of social science based on economic theory, because the shortcomings of experimental methods can be overcome by taking into account the human adaptive activities to natural and social factors, such as weather and policy [5]. FUE is defined and calculated by the ratio of optimal required input of fertilizer to the actual input when keeping output and other inputs constant [21].

Two methods are commonly used to evaluate FUE in the social sciences: data envelopment analysis (DEA) and stochastic frontier analysis (SFA). Angeles et al. [22] measured fresh vegetable production NUE using a DEA model in southeast Spain, and found that the efficiency was 0.927, indicating better efficiency in the use of nitrogen fertilizer. Lidia [23] analyzed the eco-efficiency of Chilean blueberry orchards by DEA and indicated that fertilizer had the lowest eco-efficiency. Alfonso and Francisco [24] estimated the environmental efficiency of agricultural production regarding fertilizer use in European countries with DEA, and showed that environmental efficiency from 2001 to 2012 had an increasing trend. Although DEA has the advantages of not requiring data dimensions and manual weighting, it cannot distinguish the impact of statistical noise, which may be serious in agricultural production; thus, it is recommended to use SFA for agriculture production [5,25]. Wu [15] estimated FUE in China with the SFA model and showed that the average FUE was only 0.333, and two-thirds of fertilizer use was excessive. Ma et al. [16] measured FUE in the Taihu Basin, China, using the translog SFA model, and indicated that the average FUE of rice production was only 0.254. Bai et al. [5] calculated the FUE of apple production in China with the SFA model and found that FUE had a regional characteristic, namely, the average FUE in Bohai Bay was higher than in the Loess Plateau region; the FUEs differed significantly by province. However, all of these studies estimated the FUE neglected the heteroscedasticity of the error terms, which may make the results inaccurate. Considering the heteroscedasticity of the inefficiency error term, Benedetti et al. [26] estimated the technical efficiency (TE) of irrigated crops in Italy and found that the heteroscedasticity was valid. Loureiro [27] analyzed the impact of health on agricultural productivity using the heteroscedastic SFA model, and showed that heteroscedasticity existed in both error terms. Thus, heteroscedasticity should be considered when calculating the FUE using the SFA model.

Many studies have further discussed the influencing factors of FUE. An education degree has a complex impact on FUE through changes in household behavior. These impacts include direct and indirect causal effects [16,22]. Most of them have considered that an education degree increased FUE. With an increasing education level, more skills and knowledge related to fertilizer use were mastered, absorbed, and used by householders [16]; subsequently, FUE increased accordingly [15,22]. Farm size also influenced fertilizer use intensity. Wu et al. [12] showed that fertilizer use intensity decreases with an increase in farm size. However, using the collected data on 300 farm households in Bangladesh, Nasrin et al. [28] analyzed the effect of education on the fertilizer use intensity of farmers with different farm sizes. The results indicated that education significantly affected small, medium, and large farms, but had no significant impact on marginal farms. Naseem and Kelly [29] found that a primary and secondary education degree had a significant positive impact on fertilizer use intensity. These may lead to the overuse of fertilizer and low efficiency. Zhang and Bai [30] and Bai et al. [5] showed that the degree of education had a significant negative impact on FUE, and the relationship between education and FUE had a U-shape.

Income also affected FUE. Based on the environmental Kuznets curve (EKC), fertilizer use will decrease with an increase in income [31], which may improve FUE. Based on the panel analysis, Wu et al. [12] pointed that per capita gross domestic product (PGDP) has a significant positive impact on fertilizer use intensity in China, and FUE increases and fertilizer use intensity decreases after the income reaches the inflection point of the EKC [32]. Naseem and Kelly [29] indicated that gross national product (GNP) per capita negatively affected fertilizer usage, which may increase FUE. Using the random Tobit regression model, Wu [15] indicated that household income had the significantly largest positive impact on FUE.

The non-agricultural employment ratio also had a complex impact on FUE. A higher non-agricultural employment ratio resulted in less labor input for agriculture production and more income, after which more fertilizers are used [33], which can lead to low FUE. Nasrin et al. [28] investigated the effect of non-agricultural income on fertilizer use in different farm size groups. The results showed that non-agricultural income can significantly stimulate all groups to use more fertilizer, and the smaller the farm size, the greater the impact. With the random panel Tobit model, Shi et al. [34] found that the non-agricultural employment ratio had a significant negative effect on FUE of wheat production in China from 1998 to 2013. However, with non-agricultural income increasing, farming income was not as important, and less fertilizer was used [35], increasing FUE [16]. Based on apple production panel data, Bai et al. [5] indicated that the non-agricultural employment ratio had a significant positive and the highest impact on FUE. Disasters also influence agricultural production and thus FUE. Avoiding or decreasing disasters can improve FUE [16]. On the analysis of FUE of apple orchards in China, Bai et al. [5] showed that the disaster ratio significantly negatively affected FUE.

Among the literature, the Tobit model has been commonly employed to analyze the influencing factors of FUE; however, it neglects provincial and regional differences. Because of the difference in geospatial location, the variables’ relationship and model structure will change accordingly [36]. Because the economic and regional resource endowments differ, fertilizer use intensity, FUE, and their effects obviously vary by geographical position and province [5,28,37]. Spatial analysis that considers various geographic factors can provide meaningful information to government agencies and concerned individuals [36,37]. In the context of the spatial effect of FUE, the geographically weighted regression (GWR) model is more suitable to estimate the factors affecting FUE than the ordinary least square (OLS) model [38], because the potential spatial differences can be assessed by the GWR model by examining the variations in the estimated parameters in different regions [39]. In a comparison between the OLS and GWR model to predict spatial characteristics of nitrate contamination, Koh et al. [37] indicated that the GWR models outperformed the OLS, and could provide undiscovered information that was not revealed in the OLS model. Zhou et al. [38] analyzed the different causes of haze in different regions in China with the GWR model and showed that the GWR estimate was better than the OLS and that the effects of different environmental protection inputs differ by regions. Using the data of 30 provinces in China from 2004 to 2012, Wu [39] showed that population size and affluence level were the main driving forces on the ecological footprint, and the GWR model was superior to the OLS model. Robinson et al. [40] found that GWR could effectively analyze the agglomeration and diffusion effects of nitrogen dioxide pollution in the United Kingdom. Wang et al. [36] explored the spatial effect of carbon dioxide (CO_2_) emission using the GWR model and showed that urbanization is a key factor in increasing CO_2_ emissions. These studies have demonstrated that the GWR model is more suitable than other models for estimating parameters in environmental studies [36,37,38,39], and spatial analysis results can provide a more accurate reference for regional decision-making.

In general, chemical fertilizer is considered the main source of environmental pollution in agriculture and has been overused. Thus, it is essential to explore FUE and its determinants for improving FUE and reducing fertilizer usage and environmental pollution. This paper calculated the FUE of agriculture production using a heteroscedastic stochastic frontier model, analyzed its spatial distribution and characteristics, and then estimated the spatial effects of factors affecting FUE using the GWR model.

## 3. Methodology and Data Source

This section first introduces the panel SFA model to estimate the FUE of agricultural production during 2007–2017 in China, and then the spatial correlation test method of FUE is presented, and finally, the GWR model for discussing the spatial effects of factors affecting FUE is described, followed by the data sources.

### 3.1. Calculation of Fertilizer Use Efficiency

Although no essential differences exist between the results of SFA and DEA methods, SFA is more advantageous for evaluating FUE in agricultural production [5,25]. Therefore, this study uses SFA to evaluate FUE. The SFA model was first proposed by Battese and Coelli [41], and has been widely used to evaluate efficiency of the environment, agriculture, industry, and economics. The general form is:

(1) $$y_{\mathrm{it}}=f\left(x_{\mathrm{it}},t,\beta \right)\;\exp\left(v_{\mathrm{it}}-u_{\mathrm{it}}\right)$$

where y denotes the output; i is the *i*-th observations (provinces); t refers to the time (year); x denotes the input vector; β denotes the parameter vector; v refers to the error and v ∼ N(0, σ^2^_v_); u represents technical inefficiency and is independent with v, and u∼ N(0^+^, σ^2^_u_).

Technical efficiency (TE) is defined as:

(2) $${\mathrm{TE}}_{\mathrm{it}}=y_{\mathrm{it}}/(f\left(x_{\mathrm{it},}t,\beta \right)\;\exp\left(v_{\mathrm{it}}\right))=\exp\left(-u_{\mathrm{it}}\right)$$

In this study, the translog production function is used because it has fewer limitations and can be regarded as a second-order extension of any logarithmic function, which is expressed as:

(3) $$\begin{array}{ll} {\;\mathrm{lny}}_{\mathrm{it}} & =\beta_{0}+\beta_{1}{\mathrm{lnfer}}_{\mathrm{it}}+\beta_{2}{\mathrm{lnla}}_{\mathrm{it}}+\beta_{3}{\mathrm{lnar}}_{\mathrm{it}}+\beta_{4}{\mathrm{lnme}}_{\mathrm{it}}\\ & +\beta_{5}{\mathrm{lnpe}}_{\mathrm{it}}+\beta_{6}t+\beta_{7}{\left({\mathrm{lnfer}}_{\mathrm{it}}\right)}^{2}+\beta_{8}{\left({\mathrm{lnla}}_{\mathrm{it}}\right)}^{2}+\beta_{9}{\left({\mathrm{lnar}}_{\mathrm{it}}\right)}^{2}\\ & +\beta_{10}{\left({\mathrm{lnme}}_{\mathrm{it}}\right)}^{2}+\beta_{11}{\left({\mathrm{lnpe}}_{\mathrm{it}}\right)}^{2}+\beta_{12}t^{2}+{\;\beta }_{13}{\mathrm{lnfer}}_{\mathrm{it}}{\mathrm{lnla}}_{\mathrm{it}}\\ & +{\;\beta }_{14}{\mathrm{lnfer}}_{\mathrm{it}}{\mathrm{lnar}}_{\mathrm{it}}+{\;\beta }_{15}{\mathrm{lnfer}}_{\mathrm{it}}{\mathrm{lnme}}_{\mathrm{it}}+{\;\beta }_{16}{\mathrm{lnfer}}_{\mathrm{it}}{\mathrm{lnpe}}_{\mathrm{it}}+{\;\beta }_{17}{\mathrm{tlnfer}}_{\mathrm{it}}\\ & +{\;\beta }_{18}{\mathrm{lnla}}_{\mathrm{it}}{\mathrm{lnar}}_{\mathrm{it}}+\beta_{19}{\mathrm{lnla}}_{\mathrm{it}}{\mathrm{lnme}}_{\mathrm{it}}+\beta_{20}{\mathrm{lnla}}_{\mathrm{it}}{\mathrm{lnpe}}_{\mathrm{it}}+\beta_{21}{\mathrm{tlnla}}_{\mathrm{it}}\\ & +\beta_{22}{\mathrm{lnar}}_{\mathrm{it}}{\mathrm{lnme}}_{\mathrm{it}}+\beta_{23}{\mathrm{lnar}}_{\mathrm{it}}{\mathrm{lnpe}}_{\mathrm{it}}+\beta_{24}{\mathrm{tlnar}}_{\mathrm{it}}+\beta_{25}{\mathrm{lnme}}_{\mathrm{it}}{\mathrm{lnpe}}_{\mathrm{it}}\\ & +\beta_{26}{\mathrm{tlnme}}_{\mathrm{it}}+\beta_{27}{\mathrm{tlnpe}}_{\mathrm{it}}+v_{\mathrm{it}}-u_{\mathrm{it}} \end{array}$$

where y_it_ represents agricultural output value; i = 1,2, …, 31 and refers to the provinces in China; t = 1,2, …, 18 and is the years from 1997 to 2017, which denotes technical progress for capturing the movement of the production frontier; fer_it_ represents chemical fertilizer; la_it_ represents labor; ar_it_ represents land input area; me_it_ represents total agricultural mechanical power; and pe_it_ is the pesticide input.

FUE is measured, by the definition of social science, as the ratio of the optimal required input of fertilizer to the actual input, and can be symbolized as follows [21]:

(4) $$\mathrm{FUE}=\left\{\min\left[\theta ;f\left(x,\theta \mathrm{fer};\beta \right)\geq y\right]\right\}\;\leq 1$$

where $f\left(x,\theta \mathrm{fer};\beta \right)$ represents the production frontier function; θ represents the ratio of the optimal to actual fertilizer input; x is the input vector other than fertilizer; and β denotes the parameter vector. It can be proved that the fertilizer use is effective when the technical efficiency has no loss, that is, when *u_it_ = 0*; θ_it_fer_it_ can be used instead of fer_it_ to produce the same output [21], and then Equation (3) can be derived as:

(5) $$\begin{array}{ll} {\;\mathrm{lny}}_{\mathrm{it}} & =\beta_{0}+\beta_{1}{\ln\theta }_{\mathrm{it}}{\mathrm{fer}}_{\mathrm{it}}+\beta_{2}{\mathrm{lnla}}_{\mathrm{it}}+\beta_{3}{\mathrm{lnar}}_{\mathrm{it}}+\beta_{4}{\mathrm{lnme}}_{\mathrm{it}}+\beta_{5}{\mathrm{lnpe}}_{\mathrm{it}}+\beta_{6}t\\ & +\beta_{7}{\left({\ln\theta }_{\mathrm{it}}{\mathrm{fer}}_{\mathrm{it}}\right)}^{2}+\beta_{8}{\left({\mathrm{lnla}}_{\mathrm{it}}\right)}^{2}+\beta_{9}{\left({\mathrm{lnar}}_{\mathrm{it}}\right)}^{2}+\beta_{10}{\left({\mathrm{lnme}}_{\mathrm{it}}\right)}^{2}\\ & +\beta_{11}{\left({\mathrm{lnpe}}_{\mathrm{it}}\right)}^{2}+\beta_{12}t^{2}+{\;\beta }_{13}{\ln\theta }_{\mathrm{it}}{\mathrm{fer}}_{\mathrm{it}}{\mathrm{lnla}}_{\mathrm{it}}+{\;\beta }_{14}{\ln\theta }_{\mathrm{it}}{\mathrm{fer}}_{\mathrm{it}}{\mathrm{lnar}}_{\mathrm{it}}\\ & +{\;\beta }_{15}{\ln\theta }_{\mathrm{it}}{\mathrm{fer}}_{\mathrm{it}}{\mathrm{lnme}}_{\mathrm{it}}+{\;\beta }_{16}{\ln\theta }_{\mathrm{it}}{\mathrm{fer}}_{\mathrm{it}}{\mathrm{lnpe}}_{\mathrm{it}}+{\;\beta }_{17}{\mathrm{tln}\theta }_{\mathrm{it}}{\mathrm{fer}}_{\mathrm{it}}\\ & +{\;\beta }_{18}{\mathrm{lnla}}_{\mathrm{it}}{\mathrm{lnar}}_{\mathrm{it}}+\beta_{19}{\mathrm{lnla}}_{\mathrm{it}}{\mathrm{lnme}}_{\mathrm{it}}+\beta_{20}{\mathrm{lnla}}_{\mathrm{it}}{\mathrm{lnpe}}_{\mathrm{it}}+\beta_{21}{\mathrm{tlnla}}_{\mathrm{it}}\\ & +\beta_{22}{\mathrm{lnar}}_{\mathrm{it}}{\mathrm{lnme}}_{\mathrm{it}}+\beta_{23}{\mathrm{lnar}}_{\mathrm{it}}{\mathrm{lnpe}}_{\mathrm{it}}+\beta_{24}{\mathrm{tlnar}}_{\mathrm{it}}+\beta_{25}{\mathrm{lnme}}_{\mathrm{it}}{\mathrm{lnpe}}_{\mathrm{it}}\\ & +\beta_{26}{\mathrm{tlnme}}_{\mathrm{it}}+\beta_{27}{\mathrm{tlnpe}}_{\mathrm{it}}+v_{\mathrm{it}}-u_{\mathrm{it}} \end{array}$$

Subtracting Equation (3) from (5), we obtain:

(6) $$\begin{array}{c} ({\beta \;}_{1}+{\beta \;}_{13}{\mathrm{lnla}}_{\mathrm{it}}+{\beta \;}_{14}{\mathrm{lnar}}_{\mathrm{it}}+{\beta \;}_{15}{\mathrm{lnme}}_{\mathrm{it}}+{\beta \;}_{16}{\mathrm{lnpe}}_{\mathrm{it}}\\ +{\beta \;}_{17}t)\left({\ln\theta }_{\mathrm{it}}{\mathrm{fer}}_{\mathrm{it}}-{\mathrm{lnfer}}_{\mathrm{it}}\right)+{\beta \;}_{7}[{\left({\ln\theta }_{\mathrm{it}}{\;\mathrm{fer}}_{\mathrm{it}}\right)}^{2}-\left[{\left({\mathrm{lnfer}}_{\mathrm{it}}\right)}^{2}\right]+u_{\mathrm{it}}\\ =0\; \end{array}$$

According to the definition of FUE, we have:

(7) $${\;\mathrm{lnFUE}}_{\mathrm{it}}={\ln\theta }_{\mathrm{it}}=\ln(\theta_{\mathrm{it}}{\mathrm{fer}}_{\mathrm{it}}/{\mathrm{fer}}_{\mathrm{it}})={\;\ln\theta }_{\mathrm{it}}{\mathrm{fer}}_{\mathrm{it}}-{\mathrm{lnfer}}_{\mathrm{it}}$$

FUE can be obtained in Equation (6) as:

(8) $${\;\mathrm{FUE}}_{\mathrm{it}}=\exp\left\{\left(-\lambda_{\mathrm{it}}\pm \sqrt{\lambda_{\mathrm{it}}{}^{2}-4\beta_{7}u_{\mathrm{it}}}\right)/2\beta_{7}\right\}$$

where

(9) $$\lambda_{\mathrm{it}}=\frac{\partial {\mathrm{lny}}_{\mathrm{it}}}{\partial {\mathrm{lnfer}}_{\mathrm{it}}}=\beta_{1}+\beta_{13}{\mathrm{lnla}}_{\mathrm{it}}+{\beta \;}_{14}{\mathrm{lnar}}_{\mathrm{it}}+{\beta \;}_{15}{\mathrm{lnme}}_{\mathrm{it}}+{\beta \;}_{16}{\mathrm{lnpe}}_{\mathrm{it}}+{\beta \;}_{17}t+2{\beta \;}_{7}{\mathrm{lnfer}}_{\mathrm{it}}$$

Considering that fertilizer use efficiency is in the range (0, 1), the solution out of the range of Equation (8) should be abandoned [21].

The spatial heteroscedasticity of the stochastic variance is considered (west, central, or east China), which can be introduced by different agri-ecological and soil characteristics, climate, etc. The exponential form of heteroscedasticity is used as:

(10) $$\sigma_{\mathrm{vit}}^{2}=\exp\left(\delta_{11}{\mathrm{west}}_{\mathrm{it}}+\delta_{12}{\mathrm{central}}_{\mathrm{it}}+\delta_{13}{\mathrm{east}}_{\mathrm{it}}\right)$$

where west represents whether the province is located in the western part of China, central indicates whether the province is located in the central part of China, and east shows whether the province is located in the eastern part of China. These variables represent the different agricultural production condition and resource endowment in different provinces, which are out of the farmers’ control and can greatly affect agricultural production.

The heteroscedasticity of the efficiency usually is considered to be affected by provincially specific characteristics. In this paper, the education level (edu), non-agricultural employment (non), income (inc), and disaster (dis) are assumed to affect the inefficiency variance, which is modeled as

(11) $$\sigma_{\mathrm{uit}}^{2}=\exp\left(\delta_{21}{\mathrm{edu}}_{\mathrm{it}}+\delta_{22}{\mathrm{non}}_{\mathrm{it}}+\delta_{23}{\mathrm{inc}}_{\mathrm{it}}+\delta_{24}{\mathrm{dis}}_{\mathrm{it}}\right)$$

### 3.2. Spatial Correlation Test

The spatial correlation should be tested before the GWR model. Two methods have been used to measure spatial correlation, namely, the global and local spatial correlation tests, mainly measured by Moran’s I and Geary’s C index. Moran’s I has been more widely used because it is less susceptible to deviation from the normal distribution than Geary’s C index [42]. Thus, this study employs Moran’s I to measure the correlation of FUE, and it can be measured as follows:

(12) $${\mathrm{Moran}}^{\prime }s\;I=\frac{n\sum_{i=1}^{n}\sum_{j=1}^{n}w_{\mathrm{ij}}\left(y_{i}-\bar{y}\right)\left(y_{j}-\bar{y}\right)}{\sum_{i=1}^{n}\sum_{j=1}^{n}w_{\mathrm{ij}}\sum_{i=1}^{n}{\left(y_{i}-\bar{y}\right)}^{2}}$$

where y_i_ (y_j_) denotes the observation of the *i*-th (*j*-th) province, and n denotes the number of provinces. In this study, the FUE of agriculture production is represented by y_i_, and w_ij_ represents the spatial weight matrix; $\bar{y}$ is the mean of y_i_ or y_j_ (*i*, *j* = 1, 2,…, *n*).

Moran’s I index has a value range of (−1, 1). If Moran’s I > 0, the FUE of agricultural production has a spatially positive correlation; if Moran’s I < 0, FUE has a spatially negative correlation. If Moran’s I = 0, there is no spatial correlation between FUE.

Although the global Moran’s I can analyze the spatial autocorrelation of FUE in China as a whole, it cannot specifically reflect the local spatial dependence of each province. Calculation of the local Moran’s I can obtain the spatial dependence of regional agglomeration and diffusion, and the Moran scatter plot. The scatter plot comprises four quadrants, representing different local spatial agglomeration patterns.

### 3.3. Geographical Weighted Regression Model

If the spatial correlation test is significant, the spatial model is necessary for further analysis. The GWR model has been widely used to analyze the spatial differences between different spatial locations, which was firstly proposed by Brunsdon et al. [43]. The GWR model makes it easy to check and identify patterns by measuring spatial dependence between variables [36,44]. Because of the significant provincial differences in natural resources, environments, and economic and agriculture development in China, fertilizer use intensity and FUE also have significant provincial differences [5], and the spatial differences in FUE in different provinces may lead to spatial agglomeration and diffusion. Thus, this paper employs the GWR model to examine the spatial relationship between FUE and its determinants in agricultural production. Notably, GWR is a spatial regression model [45], and is expressed as:

(13) $$y_{i}=\beta_{0}\left(\mu_{i},\upsilon_{i}\right)+\underset{k}{\sum }\beta_{k}\left(\mu_{i},\upsilon_{i}\right)x_{ik}+\varepsilon_{i}$$

where *y_i_* denotes the dependent variable for the *i*-th province; $x_{ik}$ represents the *k*-th independent variable; $\left(\mu_{i},\upsilon_{i}\right)$ is the spatial coordinates for province *i*; $\beta_{k}\left(\mu_{i},\upsilon_{i}\right)$ represents the *k*-th regression coefficient for province *i*; and $\varepsilon_{i}$ denotes the error.

To estimate the regression coefficients of each province, the spatial weight matrix must be calculated. The Gaussian kernel function is generally used to construct weights, which is:

(14) $$w_{\mathrm{ij}}=\exp\left[-{\left(\frac{d_{\mathrm{ij}}}{b}\right)}^{2}\right]$$

where w_ij_ denotes the weight for *j*-th province in the model estimated for *i*-th province, d_ij_ indicates the distance between the *i*-th and *j*-th province, and b represents the kernel bandwidth. To identify the optimal bandwidth, the cross-validation method (CV) proposed by Bowman [46] has been widely used, which is:

(15) $$CV=\overset{n}{\underset{i=1}{\sum }}{\left[y_{i}-y_{\neq i}\left(b\right)\right]}^{2}$$

where $y_{\neq i}\left(b\right)$ denotes the estimated value of y_i_. The *b* is optimal when CV is the minimum. However, different weighting methods can produce different bandwidths. Akaike information criterion (AIC) is commonly used as a criterion for determining the optimal bandwidth.

In this study, education level, income, non-agricultural employment, and disasters are used to explore the spatial impacts on FUE using the GWR model. As aforementioned in the literature review, farmers’ income is increasing rapidly with the high-speed development of the economy in China, which is a key factor influencing the FUE of agriculture production. Additionally, with the acceleration of urbanization and the non-agricultural employment of rural laborers, the non-agricultural employment ratio increase is one of the leading factors that affects FUE. The natural disaster ratio in agriculture production may also influence fertilizer use intensity and further FUE. Meanwhile, with the increase of education level and environmental awareness of farmers, a study on whether they play a critical role in China’s FUE is worthwhile. The GWR model requires variables with low correlation, and if a high correlation between the independent variables is observed, an inaccurate estimation may occur. Therefore, this paper attempts to find out the suitable variables from the main factors affecting FUE. Multicollinearity should be tested between these variables, including farmers’ age, education, income, non-agricultural employment ratio, land size, disaster ratio, irrigation fee, fertilizer price, and agriculture subsidy. Serious multicollinearity is observed among these variables. Farmers’ education level, income, non-agricultural employment ratio, and disaster ratio have a lower correlation with the variance inflation factor (VIF) test. This paper examines the spatial impacts of farmers’ education level (edu), income (inc), non-agricultural employment ratio (non), and disaster ratio (dis) on FUE. The model is:

(16) $$FUE_{i}=\beta_{0}\left(\mu_{i},\upsilon_{i}\right)+\beta_{1}\left(\mu_{i},\upsilon_{i}\right)edu_{i}+\beta_{2}\left(\mu_{i},\upsilon_{i}\right)inc_{i}+\beta_{3}\left(\mu_{i},\upsilon_{i}\right)non_{i}+\beta_{4}\left(\mu_{i},\upsilon_{i}\right)dis_{i}+\varepsilon_{i}\;$$

where $\left(\mu_{i},\upsilon_{i}\right)$ represents the spatial coordinates for province *i* (*i* = 1, 2, …, 31); *Β_k_* (k = 1,2,3,4) represents the *k*-th regression coefficient for province *i*; *FUE* denotes fertilizer use efficiency; and *Ε_i_* is the residual.

### 3.4. Data Sources

This study firstly uses the latest data for 11 years in 31 provinces of China, from 2007 to 2017, to examine the spatial differences of FUE in agriculture production, and then uses the cross-sectional data on 2007, 2010, 2014, and 2017 to explore the spatial effect of FUE and its determinants, and further analyze the changing trends of four variables during the study period.

The variables to calculate FUE, including agricultural output value, fertilizer, labor, pesticide, planting area, and mechanical inputs, are collected from the China Rural Statistical Yearbook (2008–2018) (National Bureau of Statistics of China, 2008–2018). The influencing factor data on farmers’ income, disaster ratio, and education level are obtained from the China Statistical Yearbook (2007, 2010, 2014, and 2017) (National Bureau of Statistics of China 2007, 2010, 2014, and 2017). The data on non-agricultural employment ratio comes from the China Agricultural Statistical Yearbook (2007, 2010, 2014, and 2017) (National Bureau of Statistics of China 2007, 2010, 2014, and 2017). The data have been used in many other studies [5,36], and were collected through a three-stage random sampling in the sample counties, villages, and households in each province, and then the data collected from the households selected from the samples are estimated to the provincial level data. Further, the data are tested for reliability by Cronbach’s α, which is 0.923, showing better internal consistency and stability. In addition, in order to eliminate inflation, the rural consumer price index has been used to smooth agricultural output and farmers’ income.

## 4. Results

### 4.1. Model Choice

The results of the SFA depended largely on the specific function form; thus, the specific form of the function should be tested first. The results tested with the likelihood ratio (LR) are shown in Table 1. As seen from Table 1, at a 5% significance level, the hypothesis that the translog production function would degenerate into a Cobb–Douglas (C–D) function was rejected. The following two hypothesis tests indicated that technical progress existed and was not neutral [5]. The fourth hypothesis was accepted, implying that the variances of the stochastic error in different physical locations of the provinces had no statistically significant difference, with regard to Eastern, Central and Western China (Southern and Northern China were also tested, but results were insignificant), while the last hypothesis showed that there was heteroscedasticity in the inefficiency error. Thus, the translog production function considering the heteroscedasticity of the inefficiency error and containing time and interaction with other variables was preferred for estimation.

### 4.2. Estimation Results of SFA 

The results in Table 2 show that the fixed effects model was better than the random effects model using the Hausman test. Therefore, the fixed effects stochastic frontier production function was estimated using the maximum likelihood method with Stata 12.0 (StataCorp, College Station, USA), and the results are presented in Table 2.

As shown in Table 2, the coefficients of labor and pesticide were significantly positive at the significance level of 1%, indicating that they had a positive effect on agricultural production, which was consistent with Wu [15] and Ma et al. [16]. The coefficient of mechanical power was significantly negative, but the quadratic coefficient was significantly positive, showing the overuse of mechanical power, and it had a significant U-shaped impact on agricultural production. The significantly positive coefficients of time and its quadratic indicate that technical progress existed during the study period, and its marginal impact was strengthening. Additionally, the coefficients of the interaction of time with fertilizer, labor, mechanical power, and pesticide were all significant, meaning that the technical progress was not neutral. The interaction coefficients of fertilizer and mechanical power, fertilizer and pesticide, and labor and mechanical power were all significantly negative, suggesting that a substitute relationship exists between them, which is supported by Ma et al. [16]. In addition, it can be seen from the interaction coefficients that labor and fertilizer, cropland area, and mechanical power, as well as mechanical power and pesticide, had a significant complementary relationship, which is consistent with Bai et al. [47].

In addition, farmers’ income and the non-agricultural employment ratio had a significant positive impact on the inefficiency variance, indicating that farms with higher income and more non-agricultural employment opportunities are more different in efficiency than farms with lower levels in these items. Due to the imbalance of farmers’ income and non-agricultural employment opportunities, the inefficiency variability may increase. This result is consistent with Loureiro [27], who found that the main farm operator, who works outside of the farm, has a positive impact on inefficiency variance, as this means more non-agricultural employment opportunities and income.

The TE can be calculated using Equation (2) according to the SFA model. The mean, minimum, and maximum TE values are presented in Table 2. The results show that the average TE of agricultural production was 0.816 in China from 2007–2017, and had a large variability ranging from 0.163 to 0.983. This result indicates that farmers get approximately 81.6% of the potential output by using the given inputs, and another 18.4% can be increased further under the same inputs and technical conditions if the technical inefficiency is eliminated. The result is similar to Ma et al. [16] and Singbo et al. [48]. Ma et al. [16] showed that the average TE of rice production in the Taihu Basin, China, was 0.84, and Singbo et al. [48] indicated that the average TE of vegetable production was 0.85 in Benin. These results are slightly higher than our results, probably because the heteroscedasticity in the inefficiency error term was ignored in their studies.

### 4.3. Results of FUE

Table 3 illustrates the detailed annual results of FUE in each province of China from 2007 to 2017, which is calculated by using Equation (7). As seen in Table 3, the average FUE of agricultural production was 0.722 during the research period, ranging from 0.018 to 0.989, which shows that the differences in FUE among the provinces were larger. This result is similar to Wang et al. [49], who indicated that the average FUE of agricultural production was 0.731 from 1998 to 2012 in China. FUE indicates that the fertilizer input in agriculture production in China can be decreased by 27.8% to maintain the agricultural output value with current production technologies and other inputs, and shows great potential for improved FUE and reductions in fertilizer use in China.

FUE had a declining trend over the research period. The average FUE in China decreased from 0.857 in 2007 to 0.357 in 2017 with an annual decline rate of 4.169%. The provinces of Jilin, Heilongjiang, Ningxia, Jiangsu, Inner Mongolia, Hubei, and Hainan had the fastest rate of decline. The possible reason for this finding is that the usage of chemical fertilizer has increased significantly, but the agricultural output value has not increased much in China, resulting in the overuse of fertilizer and lower FUE [5,16,33].

**Figure 1:**
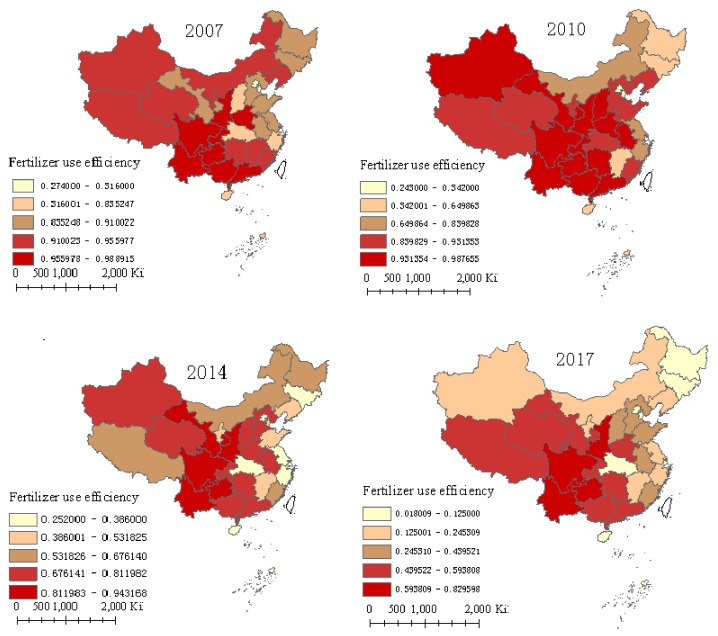
Fertilizer use efficiency in 2007, 2010, 2014, and 2017 in China.

Figure 1 presents the spatial distribution of FUE in each province in 2007, 2010, 2014, and 2017. The FUE shows obvious spatial differences, whereas the relative position of FUE in each province changed slightly in different years. On the whole, four provinces, including Shaanxi, Sichuan, Guizhou, and Yunnan, had the highest FUE, with an average FUE of more than 0.910, located in the first echelon. Three municipalities, including Beijing, Tianjin, and Shanghai, had the lowest FUE, and ranked in the last echelon. The relative spatial position of the FUE in each province remained basically stable during the study period. Compared with 2007, the relative position of Gansu has made rapid progress, whereas the relative positions of Guangdong, Guangxi, and Henan regressed after 2014. The obvious spatial disequilibrium of FUE among provinces and regions may be related to the wider agricultural production areas. China’s agricultural production covers different regions, such as tropical, subtropical, and temperate zones, and the different climatic conditions, resource endowments, planting habits, and economic developments in different regions have led to significant differences in fertilizer use and efficiency [5,15,33].

Figure 1 also presents the spatial agglomeration effect of FUE. The provinces with higher FUE are mainly distributed in Southwest China, while the provinces with lower FUE were mainly concentrated in Central and Eastern China, except for the Henan and Guangdong provinces. FUEs have adjacent spatial agglomeration characteristics of high–high efficiency and low–low efficiency, which may be related to the similar resource endowments and economic development in neighboring regions. On the one hand, because of the similarity of resource endowments in adjacent regions, the production behaviors of farmers in adjacent regions are roughly the same, for example, the convergence of crop types and the similarity of input factors, leading to a certain spatial dependence of FUE. Moreover, according to technology diffusion theory, agricultural technology will preferentially spill over to neighboring areas with similar conditions, leading to spatial correlation. On the other hand, similar economic development in adjacent areas also affects FUE. Because of the relatively low comparative income of agricultural production, farmers in the regions with higher economic development tend to cultivate on a large scale, which is conducive to exerting the scale effect of land and reducing the usage of chemical fertilizers [12]; by contrast, farmers in regions with low economic development, such as the Hubei and Jiangxi provinces, tend to transfer labor to non-agricultural industries with higher income. The reduction in labor will reduce the field management intensity of farmers, but the increase in fertilizer can make up for the loss of labor to a certain extent, which leads to increased fertilizer use to replace labor [28,33], resulting in the correlation between FUE and regional economic development. Therefore, because of the similarity of resource endowments and production behaviors in the neighboring regions, as well as the spatial spillover effects of production skills, FUE has spatial convergence and agglomeration in adjacent regions.

### 4.4. Spatial Autocorrelation Test of FUE

Based on the qualitative analysis of spatial autocorrelation of FUE in the previous section, the Moran’s I index will be further used for quantitative analysis. Table 4 shows the results of the global Moran’s I index for FUE. The Moran’s I index in each year was significantly greater than zero and showed an increasing trend. Thus, FUE had a significant positive spatial correlation in different provinces, and the correlation was increasing.

**Figure 2:**
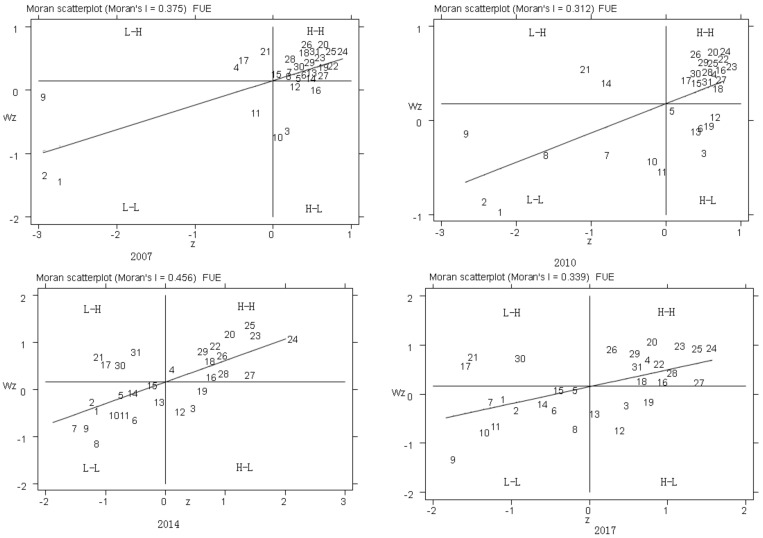
Moran’s I scatter plot of fertilizer use efficiency in 2007, 2010,2014, and 2017.

Furthermore, the Moran scatter plots were used to test the spatial correlation and agglomeration of each province (Figure 2). There were 24 provinces distributed in the first and third quadrants in 2007; among them, six provinces in the eastern areas, such as Shandong, Fujian, Guangdong, Liaoning, Jilin, Heilongjiang, two provinces in Central China, including Jiangxi and Hunan, and all of the twelve provinces in Western China were the H–H agglomeration type. Another four provinces, including Beijing, Tianjin, Shanghai, and Zhejiang, were the L–L agglomeration type. This result indicates that FUE in these provinces had a high positive spatial correlation. Three provinces, namely, Shanxi, Hubei, and Hainan, were the L–H agglomeration type, and four provinces, namely, Hebei, Jiangsu, Anhai, and Henan, were the H–L agglomeration type, indicating a negative spatial correlation between FUE in these provinces. Additionally, the quadrant positions of the provinces and autonomous areas in the scatter plots were relatively stable from 2007 to 2010, but a few quadrant jumps were observed in 2014; three provinces, including Hubei, Ningxia, and Xinjiang, have jumped from the first quadrant to the second quadrant. Meanwhile, the provinces of Inner Mongolia, Liaoning, and Fujian have jumped from the fourth quadrant to the third quadrant, and then stabilized from 2014 to 2017. Overall, FUE had a strong and stable spatial correlation and showed a spatial agglomeration effect. Thus, it is necessary to analyze the spatial effect of FUE using the GWR model.

### 4.5. Results of GWR 

The GWR model requires variables with a low correlation; thus, an assessment of the multicollinearity of variables before using the GWR model is required. The VIF was used to test the multicollinearity of the variables, and the result showed that the VIF was no more than 7.5, showing no multicollinearity among the variables [36].

Table 5 demonstrates the regression result of factors affecting FUE in 2007, 2010, 2014, and 2017, which was measured using ArcGIS 10.5. Adjusted R^2^ values show spatial variation throughout different provinces; it had the largest value of 0.574 in 2010 and had the smallest value of 0.414 in 2017. Additionally, the adjusted R^2^ value demonstrated a downward trend with the increase in years.

Table 5 also illustrates the maximum and minimum coefficients of 31 provinces over four years. The result shows that farmers’ education level, non-agricultural employment ratio, disaster ratio, and income had stronger explanatory power on FUE. The farmers’ education level and income had a negative impact on FUE, and the effect of farmers’ income on FUE was significant at the level of 1%, whereas the non-agricultural employment ratio had a significant positive impact on FUE, and the impact of the disaster ratio on FUE was uncertain, which may mainly depend on the degree of natural disaster. Among the influencing factors, the non-agricultural employment ratio had the highest impact on FUE compared to the other three factors. Specifically, the smallest coefficient of education level in 2007 was −0.002, and then it decreased to −0.042 in 2017, with a clear downward trend. Regarding the non-agricultural employment ratio, the coefficients were 0.109, 0.525, 0.278, and 0.601 over the four years, indicating that the impact of the non-agricultural employment ratio on FUE increased in fluctuation with the increasing years. The impact of the disaster ratio on FUE had the characteristic of an inverted U-shape. The greatest coefficients of farmers’ income were −0.087, −0.087, −0.051, and −0.043 over the four years, indicating that farmers’ income had an increasing impact on FUE.

## 5. Discussion

### 5.1. Education Level

**Figure 3:**
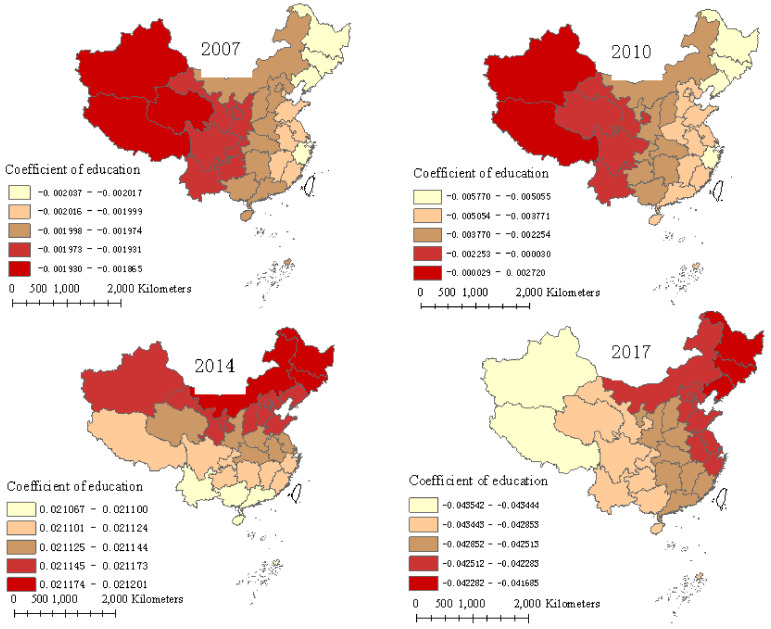
Regression coefficients of education over four years.

Figure 3 reveals the effect of education level on FUE in the 31 provinces. The result shows that the impact of education level on FUE was negative in the studied years, except for 2014, which is consistent with Bai et al. [5]. Compared with other factors, the education level has a relatively weak impact on FUE, which is supported by the results of Wu [15], who found that education had the smallest effect on FUE. Additionally, the difference of coefficients among neighboring provinces was small, but the regression coefficients had an obvious spatial difference in the eastern, central, and western areas, declining from the western to eastern areas in 2007 and 2010 and decreasing from northeastern to southwestern areas in 2014 and 2017, indicating that the education level had a significant spatial dependence on the impact of FUE. Specifically, the most affected provinces were Xinjiang and Tibet in 2007 and 2010, whereas the less affected provinces were concentrated in eastern and northeastern areas, such as the Liaoning, Jilin, Heilongjiang, and Zhejiang provinces. The greatest affected provinces were Heilongjiang and Jilin in 2014 and 2017, and the least affected provinces were concentrated in southwestern areas, including Tibet and Yunnan. The average regression coefficient of education level was −0.002, −0.003, 0.021, and −0.043 in 2007, 2010, 2014, and 2017, respectively, indicating that the influence degree increased by year.

Although education level has an insignificant effect on FUE, the regression coefficient shows that the main impact of education level on FUE is negative, which is consistent with Zhang and Bai [30] and Bai et al. [5]. On the one hand, a higher education level means a higher awareness of the role of fertilizer, which may lead to greater reliance on fertilizer as an input [33], and more fertilizer use may lead to lower FUE. On the other hand, a higher education level means more non-agricultural employment opportunities and higher agricultural labor opportunity costs; thus, a greater number of chemical fertilizers will be used to replace the labor force [5], resulting in lower FUE. Education level had a positive impact on FUE in 2014, which may be correlated with the economic development situation of China in 2014. In 2014, economic development was not optimistic in China, and farmers decreased agricultural production inputs, including reducing the fertilizer input, which may have improved FUE. In addition, education level has a different spatial impact on FUE, which may be caused by the unbalanced education in China, indicating that the marginal effect of increasing education level on FUE is different.

Therefore, in order to improve FUE and, at the same time, solve the problem of uneven education, the government should focus on strengthening training for farmers on fertilizer use techniques, such as formula fertilizer, water, and fertilizer integration, etc., stressing the environmental awareness of fertilizer use and encouraging the reduction of fertilizer use, improving the comparative advantage of agriculture and reducing the substitution effect of chemical fertilizer on labor.

### 5.2. Non-Agricultural Employment Ratio

**Figure 4:**
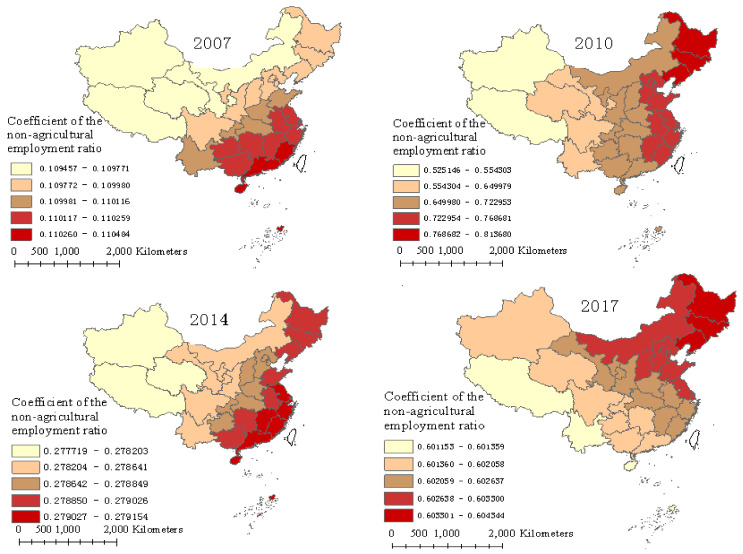
Regression coefficients of the non-agricultural employment ratio over four years.

As shown in Figure 4, the non-agricultural employment ratio was the leading factor in improving FUE in each year. The average regression coefficient of the non-agricultural employment ratio on FUE in each province shows an N-shaped fluctuation characteristic from 2007 to 2017, with an average value of 0.424 and a range from 0.109 in 2007 to 0.604 in 2017. In general, the non-agricultural employment ratio had a great impact on FUE in the southeastern coastal provinces, for example, Fujian, Zhejiang, and Jiangsu, and had a low impact on the western provinces, such as Xinjiang, Tibet, and Qinghai. The degree of influence of the non-agricultural employment ratio on FUE shows a decreasing trend from the southeastern to northwestern provinces in 2007 and 2014, while it shows a decreasing trend from eastern to western provinces in 2010 and from northeastern to southwestern provinces in 2017. Generally, the spatial difference of effect in different provinces was very small over the study years, and the main difference was between the eastern, central, and western provinces, which is consistent with the degree of economic development and economic situation in China.

The major mechanisms for the non-agricultural employment ratio to increase FUE rely on three aspects. As the non-agricultural employment ratio increases and the agricultural production labor decreases, more machines, fewer and higher quality fertilizers are used to replace labor, which is conducive to improving FUE. Additionally, increased non-agricultural employment tends to accelerate land transfer. Subsequently, the scale economy induced by land transfer reduces the use of chemical fertilizers, improving FUE. In addition, the higher the non-agricultural employment ratio, the higher the income, the stronger the environmental awareness, and the less dependence on agriculture, which results in less fertilizer used [12]; thus, it is conducive to the improvement in FUE. Therefore, the effect of the non-agricultural employment ratio on FUE in various regions shows a trend consistent with the economic level of each region in general.

### 5.3. Disaster

**Figure 5:**
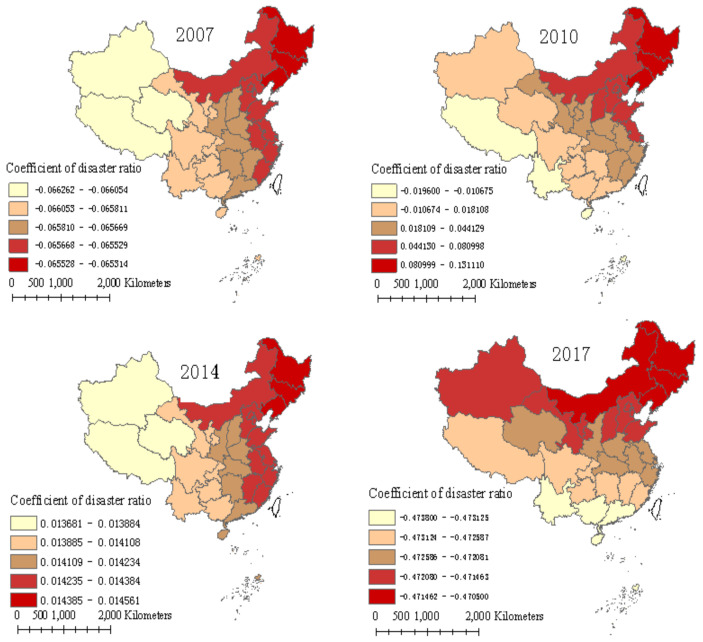
Regression coefficients of the disaster ratio over four years.

Figure 5 reflects the effect of the disaster ratio on FUE in 31 provinces from 2007 to 2017. The greatest regression coefficients of the disaster ratio were −0.065, 0.131, 0.015, and −0.471 in 2007, 2010, 2014, and 2017, respectively. This finding suggests that the effect of the disaster ratio on FUE was unstable. In 2007, the influence of the disaster ratio was negatively correlated with FUE, indicating that the decrease in the disaster ratio was conducive to the improvement in FUE. The provinces most affected by the disaster ratio were mainly located in the northeast of China, for example, Liaoning, Jilin, and Heilongjiang, and the least affected provinces were Xinjiang, Qinghai, and Tibet. The impact had an obvious declining trend from the east to the middle to the west.

However, in 2010, the influence of the disaster ratio on FUE was complicated. In some provinces, such as Xinjiang, Tibet, Yunnan, and Hainan, the effect was negative, while in the other provinces, the effect of the disaster ratio on FUE was positive, and the impact decreased from the northeastern to the southwestern regions. The opposite impact of the disaster ratio on FUE may be related to the severity of the disaster. On the one hand, if the disaster is too severe to recover from, farmers will reduce the excessive fertilizer input to reduce the irreparable losses, which may improve FUE; on the other hand, if the disaster is not serious, farmers increase fertilizer input to reduce the recoverable losses [18], decreasing FUE [16]. The impact of the disaster ratio on FUE was positive in 2014, and the regions most influenced were mainly concentrated in northeastern China, including Heilongjiang, Jilin, and Liaoning. In 2017, the impact was negative and decreased from the northeastern to the southwestern regions. Overall, the impact of the disaster ratio on FUE decreased from east to west in 2007 and 2014, and decreased from the northeastern to the southwestern region in 2010 and 2017.

The different impacts of the disaster ratio on FUE may be related to the different resource endowments and adaptability of farmers to different severities and types of natural disasters [16]. Therefore, to improve FUE and reduce the disaster ratio to a manageable range, it is necessary to make the best of resource endowment advantages of various regions while using new technologies to transform production methods, for example, strengthening infrastructure construction of irrigation systems in arid and semi-arid areas and flood drainage facilities in southeastern regions that are usually affected by typhoons, and establishing meteorological disaster forecasting systems to improve the adaptive capacity to manage climate disasters.

### 5.4. Farmers’ Income

**Figure 6:**
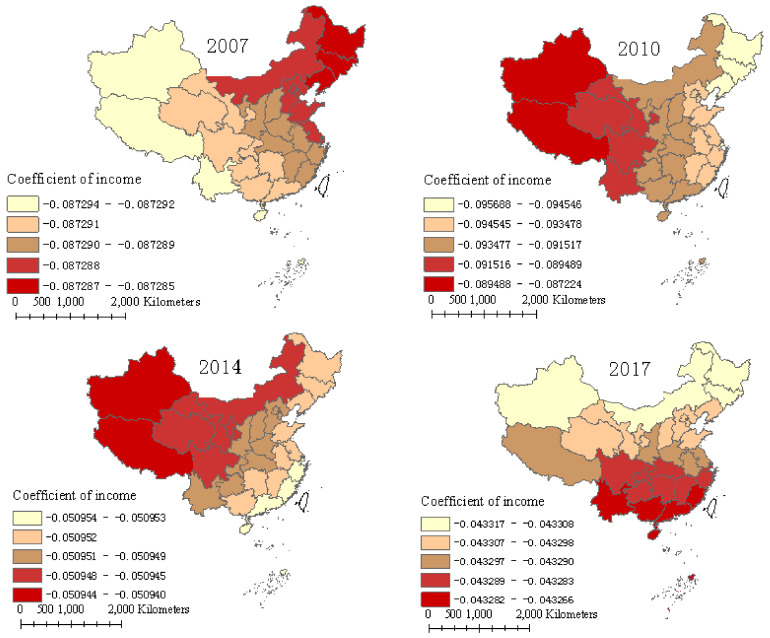
Regression coefficients of farmers’ income over four years.

In Figure 6, the largest regression coefficients of per capita income of farmers were −0.087, −0.087, −0.051, and −0.043 in 2007, 2010, 2014, and 2017, respectively, indicating that the per capita income of farmers had a negative impact on FUE, and the degree of influence decreased by year. In addition, the difference in the spatial effect of income on FUE was tiny among different regions and provinces in the studied years, except in 2010, which had an influence degree with an obvious increasing trend from the western to the eastern parts of China.

The negative effect of income on FUE could be the result of two aspects. First, the increase in farmers’ income will effectively alleviate their financial constraints, and farmers will invest in more fertilizer, leading to low efficiency [33]. Provinces in Central and Eastern China are the main agricultural production areas, and more income may lead to more fertilizer use and less efficiency, which is also supported by Figure 1. Second, as a developing country, China’s per capita income is not high. With the increase in income, the demand for food is increasing, which leads to an increase in the use of fertilizer in pursuit of food quantity safety, resulting in low FUE. Additionally, according to the EKC, as farmers’ incomes increase, their environmental awareness increases, and farmers reduce the overuse of fertilizers or apply higher-quality fertilizers that are easily absorbed or release nutrients according to the growth cycle of crop, increasing FUE. However, the excessive use of fertilizers still had a negative impact on FUE, whereas the degree of negative impact declined over time. This is supported by Ma et al. [16], who found that fake fertilizer with fewer nutrients could not reduce the yield, indicating that the fertilizer use was excessive. When income increases to the turning point of the EKC, it will benefit the improvement in FUE, reducing agricultural non-point source pollution [12,50]. Thus, to improve the FUE, in addition to increasing machinery, modern technology, and management practices, we should also focus on policy integration to increase the per capita income of farmers.

## 6. Conclusions

By calculating the FUE of agricultural production in 31 provinces from 2007 to 2017 using a translog stochastic frontier function panel model considering the heteroscedasticity of error, the spatial effects of FUE were analyzed. Based on the spatial data for 2007, 2010, 2014, and 2017, this study explored the impacts of education level, the non-agricultural employment ratio, the disaster ratio, and the per capita income of farmers on FUE, and revealed the spatial correlation using the GWR model. The result indicates that FUE had a significant spatially positive correlation. The non-agricultural employment ratio had the highest impact on FUE, and it was the leading factor for increasing FUE in all provinces in years studied and should, therefore, be applied as the main means of increasing FUE and achieving the goal of reducing fertilizer use. Farmers’ income negatively affected FUE, and its degree of influence decreased year by year. The influence of education level on FUE was negative, but relatively weak. In general, the disaster ratio had a different impact on FUE in different years. The results have notable implications for policy decisions.

(1) Due to the strong and stable spatial agglomeration effect of FUE in different provinces, policies to reduce fertilizer use and increase FUE must be formulated considering the spatial effects of provinces. The Chinese government should formulate reasonable fertilizer reduction and efficiency increase targets for all provinces, according to local conditions and natural resource endowment characteristics. For example, in South China, where the livestock industry is concentrated, the government should encourage the use of farmyard manure to replace chemical fertilizer input; in arid and semi-arid regions, water−fertilizer integration and even drip fertigation systems should be extended and subsidized [51], which can reduce fertilizer use and improve FUE. In addition, due to the difference of regional (farmer) characteristics, the heteroscedasticity of error should be considered when estimating the FUE of agriculture production, especially in regions (farmers) with high income and a high non-agricultural employment ratio, which have significant positive impacts on the heteroscedasticity of error.

(2) The influence of education level on FUE was negative, and the influence degree was increasing. Therefore, the government should strengthen practical technical training for farmers on fertilizer use while improving the level of education, such as chemical fertilizer use in furrows, water and fertilizer integration, etc., and emphasize the negative impact of fertilizers on the environment for reducing fertilizer use. In addition, due to the spatial effect of education on FUE, it was different in different provinces, and an exploration of educational level improvement and a training mechanism for different regions with local characteristics is necessary.

(3) The non-agricultural employment ratio was the leading factor increasing FUE, and should be applied as the main means of increasing FUE and achieving the goal of reducing fertilizer use. In addition, the degree of influence of the non-agricultural employment ratio had a decreasing trend from the eastern to western provinces of China. Thus, different agricultural industry development modes and policy incentive systems for increasing the non-agricultural employment ratio should be developed for different regions. For the plains areas, such as North China, which is suitable for large-scale production, the government should increase land transfer, appropriately increase mechanization subsidies, and promote agricultural production services and large-scale agricultural production, which can release labors and increase the non-agricultural employment ratio, and thus improve FUE [5,15]. Moreover, crop planting patterns should be adjusted according to the respective natural resource endowments in each region, and the development path of characteristic agriculture and green agriculture should be followed. For example, in Northeast China, the rotation of corn and soybeans should be adopted and promoted to improve soil fertilizer and reduce fertilizer use. In addition, conservation tillage technology should be promoted, which can save labor and fertilizer use and may thereby improve FUE [18].

(4) The impact of the disaster ratio on FUE was roughly not the same from 2007 to 2017, and the distribution of the degree of influence also differed. The influence of the disaster ratio on FUE was mainly related to the type and severity of the disasters [16], such as heavy wind or drought. Therefore, each region should adopt appropriate adaptive measures according to their resource endowments to deal with different types of disasters and reduce the disaster severity. The government should increase financial subsidies for regions or provinces with severe or frequent disasters, improve the agricultural insurance system, strengthen the development of rural finance, and establish a sound agricultural infrastructure, for example, irrigation and drainage facilities. Furthermore, the government should provide a timely forecast and publicize weather and possible disasters, provide farmers with disaster prevention technology, and guarantee agricultural production.

(5) The per capita income of farmers had a negative impact on FUE, but its degree of impact decreased by year. Therefore, to improve FUE, all regions or provinces should do everything possible to improve the per capita income of rural residents, such as improving non-agricultural employment opportunities, developing e-commerce, accelerating land transfer to promote the scale benefit, etc. At the same time, environmental awareness should also be strengthened.

The conclusions provide a policy reference for the government to implement fertilizer reduction and FUE increase in China. However, there are still some limitations to the study. First, the agricultural resource endowments and crop planting patterns in different provinces are usually different and will affect the FUE, but this paper does not consider the effect of crop planting patterns on FUE for each province based on the spatial effect. Second, the study reflects on the FUE of agricultural production on the whole, ignoring the possible different responses of different crops. Due to the different production characteristics of crops, e.g., cash crop and food crop, the different production methods and their responses may lead to different FUE, thus, further studies should pay more attention to the analysis of specific crops.

## Figures and Tables

**Table 1:** Model tests.

Null Hypothesis	Degree of Freedom (k)	LR Test	Threshold χ^2^_0.05_(k)	Decision
C-D production function H_0_: β_7_ = β_8_ = β_9_ = …= β_27_ = 0	21	72.174	32.670	Reject
No technical progress H_0_: β_6_ = β_12_ = β_17_ = β_21_ = β_24_ = β_26_ = β_27_ = 0	7	780.048	14.067	Reject
Neutral technical progress H_0_: β_17_ = β_21_ = β_24_ = β_26_ = β_27_ = 0	5	116.652	11.070	Reject
Heteroscedastic variance of stochastic errorH_0_: δ_1_ = 0	3	6.326	7.045	Accept
Heteroscedastic variance of inefficiency errorH_0_: δ_2_ = 0	4	70.245	8.761	Reject

**Table 2:** Estimated results of SFA.

Variable	Coefficient	Standard Error	Variable	Coefficient	Standard Error
Constant(β_0_)	−0.189	0.151	lnFer*Time(β17)	0.029 **	0.014
lnFer(β_1_)	1.506	1.334	lnLa*lnAr(β18)	−0.149	0.089
lnLa(β_2_)	3.269 ***	1.228	lnLa*lnMe(β19)	−0.822 ***	0.204
lnAr(β_3_)	−0.017	0.502	lnLa*lnPe(β20)	−0.240	0.154
lnMe(β_4_)	−2.787 ***	0.991	lnLa*Time(β21)	0.053 ***	0.014
lnPe(β_5_)	1.792 ***	0.690	lnAr*lnMe(β22)	0.221 ***	0.080
Time(β_6_)	0.309 ***	0.059	lnAr*lnPe(β23)	−0.038	0.048
lnFer*lnFer(β_7_)	−0.001	0.185	lnAr*Time(β24)	0.006	0.010
lnLa*lnLa(β_8_)	0.011	0.163	lnMe*lnPe(β25)	0.277 **	0.120
lnAr*lnAr(β_9_)	−0.021	0.064	lnMe*Time(β26)	−0.076 ***	0.013
lnMe*lnMe(β_10_)	0.416 ***	0.144	lnPe*Time(β27)	−0.016 **	0.007
lnPe*lnPe(β_11_)	0.001	0.106	Inefficiency variance (σu2)		
Time*Time(β_12_)	0.007 ***	0.001	edu	0.034	0.102
lnFer*lnla(β_13_)	1.436 ***	0.311	income	0.0003 ***	0.000
lnFer*lnAr(β_14_)	0.004	0.097	nonagr	3.118 ***	0.954
lnFer*lnMe(β_15_)	−0.582 **	0.253	disa	−0.112	0.592
lnFer*lnPe(β_16_)	−0.542 ***	0.137	constant	−3.916 ***	0.855
look likelihood		146.913			
Hausman test		Chi-square = 64.31		*p*-value = 0.000	
Mean TE 0.816		(min, max)		(0.163, 0.983)	

Note: ***, **, and * denote the significance levels of 1%, 5%, and 10%, respectively.

**Table 3:** Fertilizer use efficiency in 31 provinces of China.

Province	2007	2008	2009	2010	2011	2012	2013	2014	2015	2016	2017	Mean
Beijing	0.316	0.323	0.336	0.342	0.353	0.364	0.373	0.386	0.385	0.394	0.102	0.334
Tianjin	0.278	0.273	0.286	0.295	0.316	0.327	0.387	0.421	0.524	0.645	0.084	0.349
Hebei	0.873	0.899	0.907	0.931	0.940	0.906	0.896	0.716	0.535	0.901	0.440	0.813
Shanxi	0.765	0.749	0.947	0.963	0.962	0.926	0.865	0.761	0.566	0.682	0.382	0.779
Inner Mongolia	0.921	0.905	0.831	0.840	0.865	0.890	0.833	0.580	0.388	0.894	0.191	0.740
Liaoning	0.932	0.880	0.784	0.922	0.806	0.746	0.696	0.524	0.480	0.943	0.243	0.723
Jilin	0.899	0.896	0.770	0.650	0.663	0.716	0.439	0.362	0.137	0.822	0.018	0.579
Heilongjiang	0.897	0.718	0.718	0.474	0.682	0.685	0.808	0.578	0.225	0.848	0.101	0.612
Shanghai	0.274	0.276	0.283	0.243	0.247	0.249	0.251	0.252	0.253	0.342	0.062	0.248
Jiangsu	0.863	0.836	0.809	0.778	0.795	0.699	0.463	0.333	0.216	0.896	0.163	0.623
Zhejiang	0.812	0.862	0.810	0.808	0.832	0.577	0.462	0.351	0.280	0.871	0.192	0.623
Anhui	0.910	0.962	0.928	0.960	0.953	0.867	0.786	0.695	0.519	0.954	0.408	0.813
Fujian	0.951	0.901	0.861	0.904	0.874	0.808	0.761	0.620	0.506	0.966	0.331	0.771
Jiangxi	0.950	0.924	0.826	0.645	0.505	0.223	0.643	0.488	0.417	0.964	0.231	0.620
Shandong	0.859	0.942	0.931	0.907	0.871	0.649	0.642	0.532	0.424	0.837	0.304	0.718
Henan	0.962	0.963	0.957	0.974	0.960	0.726	0.846	0.812	0.694	0.891	0.523	0.846
Hubei	0.779	0.825	0.760	0.877	0.911	0.824	0.519	0.283	0.102	0.911	0.125	0.629
Hunan	0.938	0.940	0.899	0.969	0.961	0.933	0.839	0.755	0.671	0.963	0.519	0.853
Guangdong	0.982	0.965	0.926	0.943	0.940	0.915	0.824	0.773	0.676	0.887	0.491	0.847
Guangxi	0.978	0.970	0.914	0.956	0.945	0.896	0.850	0.783	0.731	0.982	0.594	0.873
Hainan	0.835	0.710	0.476	0.584	0.463	0.594	0.306	0.299	0.227	0.918	0.105	0.502
Chongqing	0.985	0.973	0.959	0.976	0.958	0.947	0.846	0.793	0.682	0.931	0.539	0.872
Sichuan	0.973	0.977	0.964	0.979	0.977	0.953	0.912	0.858	0.812	0.978	0.691	0.916
Guizhou	0.989	0.992	0.981	0.988	0.928	0.957	0.920	0.943	0.955	0.962	0.830	0.950
Yunnan	0.980	0.980	0.970	0.954	0.898	0.969	0.937	0.904	0.780	0.967	0.668	0.910
Tibet	0.956	0.901	0.957	0.905	0.947	0.856	0.647	0.676	0.506	0.972	0.564	0.808
Shaanxi	0.969	0.980	0.975	0.979	0.985	0.930	0.916	0.909	0.873	0.907	0.677	0.918
Gansu	0.909	0.874	0.867	0.946	0.970	0.955	0.948	0.836	0.697	0.771	0.566	0.849
Qinghai	0.948	0.952	0.877	0.927	0.970	0.922	0.805	0.736	0.567	0.983	0.494	0.835
Ningxia	0.920	0.943	0.909	0.916	0.930	0.802	0.646	0.429	0.265	0.726	0.186	0.697
Xinjiang	0.951	0.883	0.844	0.944	0.904	0.786	0.673	0.754	0.393	0.564	0.245	0.722
mean	0.857	0.844	0.815	0.822	0.816	0.761	0.701	0.618	0.500	0.847	0.357	0.722

**Table 4:** Moran’s I index of fertilizer use efficiency in China.

Index/Year	2007	2010	2014	2017
Moran’s I	0.235	0.247	0.333	0.444
*p*-value	0.003 ***	0.004 ***	0.000 ***	0.000 ***
Z-value	2.700	2.662	3.332	4.349

Note: ***denote the significance levels of 1%.

**Table 5:** GWR regression results.

Parameter	Year							
	2007		2010		2014		2017	
	Min.	Max.	Min.	Max.	Min.	Max.	Min.	Max.
Intercept	1.233719 ***	1.234685 ***	1.066785 ***	1.117555 ***	0.853885 ***	0.855233 ***	1.043769 ***	1.062382 ***
Education level	−0.002037	−0.001865	−0.005770	0.002720	0.021067	0.021201	−0.043542	−0.041685
Non-agriculture employment ratio	0.109457	0.110484	0.525146 ***	0.813680***	0.277719	0.279154	0.601153**	0.604344 **
Disaster ratio	−0.066262	−0.065314	−0.019600	0.131110	0.013681	0.014561	−0.473800 *	−0.470500 *
Farmers’ income	−0.087294 ***	−0.087285 ***	−0.095688 ***	−0.087224 ***	−0.05095 ***	−0.05094 ***	-0.043317 ***	−0.043266 ***
Bandwidth	431.058		45.520		431.058		185.083	
AIC	−28.619		−22.526		−15.936		−11.376	
Adjusted R^2^	0.571		0.574		0.423		0.414	

Note: ***, **, and * denote the significance levels of 1%, 5%, and 10%, respectively.

## Abbreviations

FUE	fertilizer use efficiency
GWR	geographical weighted regression
SFA	stochastic frontier analysis
NUE	nitrogen use efficiency
CS-PNM	canopy sensor-based precision N management
DEA	data envelopment analysis
EKC	environmental Kuznets curve
PGDP	per capita gross domestic product
TE	technical efficiency
GNP	gross national product
OLS	ordinary least square
CO2	carbon dioxide
CV	cross-validation
AIC	Akaike information criterion
VIF	variance inflation factor
C-D	Cobb–Douglas
LR	likelihood ratio
